# Historical Perspective, Development and Applications of Next-Generation Sequencing in Plant Virology

**DOI:** 10.3390/v6010106

**Published:** 2014-01-06

**Authors:** Marina Barba, Henryk Czosnek, Ahmed Hadidi

**Affiliations:** 1 Consiglio per la ricerca e la Sperimentazione in Agricoltura, Centro di Ricerca per la Patologia Vegetale, Via C. G. Bertero 22, Rome 00156, Italy; 2 Institute of Plant Sciences and Genetics in Agriculture, The Robert H. Smith Faculty of Agriculture Food and Environment, The Hebrew University of Jerusalem, Rehovot 76100, Israel; E-Mail: hanokh.czosnek@mail.huji.ac.il; 3 U.S. Department of Agriculture, Agricultural Research Service, Beltsville, MD 20705, USA; E-Mail: ahadidi@yahoo.com

**Keywords:** next-generation (deep) sequencing, NGS, novel virus/viroid discovery, metagenomics, virome, transcriptome, DNA sequencing, RNA sequencing (RNA-seq.)

## Abstract

Next-generation high throughput sequencing technologies became available at the onset of the 21st century. They provide a highly efficient, rapid, and low cost DNA sequencing platform beyond the reach of the standard and traditional DNA sequencing technologies developed in the late 1970s. They are continually improved to become faster, more efficient and cheaper. They have been used in many fields of biology since 2004. In 2009, next-generation sequencing (NGS) technologies began to be applied to several areas of plant virology including virus/viroid genome sequencing, discovery and detection, ecology and epidemiology, replication and transcription. Identification and characterization of known and unknown viruses and/or viroids in infected plants are currently among the most successful applications of these technologies. It is expected that NGS will play very significant roles in many research and non-research areas of plant virology.

## 1. Introduction

Next-generation (high throughput, deep) sequencing (NGS) has been developed in recent years. These technologies have lowered the costs of DNA sequencing beyond what is possible with standard dye-terminator methods. NGS describes platforms that produce large amounts (typically millions to billions) of DNA reads, with lengths between 25 and 400 bp. These reads are shorter than the traditional Sanger sequence reads (300 to 750 bp). Recently, however, NGS technologies have advanced to produce DNA reads longer than 750 bp. The roots of NGS development go back to the discovery of the structure of the DNA double helix in 1953 by James Watson and Francis Crick, an American geneticist and a British physicist, respectively [1]. Robert Holley, an American biochemist, was the first to sequence a nucleic acid when he and colleagues developed sequencing methods for tRNA in 1964 and 1965 [2,3]. Sequencing methods for long DNA were successfully developed independently in 1977 by Frederick Sanger, a British biochemist [4,5], and by Walter Gilbert, an American biochemist/physicist [6]. The Nobel Prize was received by Watson and Crick in 1962, by Holley in 1968, and by Sanger and Gilbert in 1980.

## 2. Pioneer Landmarks in DNA Sequencing

Several years before any attempt was made to sequence a DNA molecule, in 1964 Robert Holley (1922–1993) was the first to sequence an RNA molecule. He determined the complete sequence and structure of the 77 ribonucleotides of alanine tRNA, the molecule that incorporates the amino acid alanine into protein. Holley’s pioneer work opened the door for others to determine the sequence of other RNAs as well as DNA.

In 1972, Paul Berg (1926–present) developed DNA technology, which permits isolation of defined fragments of DNA [7]. Prior to this, the only accessible samples for sequencing were from phages or virus DNA. Also, his discovery led to the development of the modern genetic engineering.

In 1973, the first nucleotide sequence of 24 bp out of 27 bp of the *lac* operator DNA was published [8]. In 1977, Frederick Sanger (1918–2013) was the first to sequence the complete DNA genome of bacteriophage ΦX 174 [4]. He also developed “DNA sequencing with chain-terminating inhibitor” [5]. Moreover, in 1977 Walter Gilbert (1932–present) developed “DNA sequencing by chemical degradation” [6]. 

Paul Berg, Frederick Sanger and Walter Gilbert received the Nobel Prize in Chemistry in 1980. Frederick Sanger received the Noble Prize twice, the first one in 1958 for his work on the structure of proteins, especially that of insulin. Sanger is one of only four people to win two Nobel Prizes and the only Nobel Laureate to win two chemistry prizes.

## 3. Major Landmarks in DNA Sequencing during the Last Three Decades

1984: Medical Research Council scientists [9] completed the DNA sequence of the *Epstein-Barr virus* (EBV) 172,282 bp using the dideoxynucleotide/M13 sequencing procedure. EBV causes infectious mononucleosis.

1986: LeRoy Hood at the California Institute of Technology (Cal Tech., Pasadena, CA, USA) announced the invention of the first-semi-automated DNA sequencing machine. The machine automated the enzymatic chain termination procedure for DNA sequence analysis developed by Sanger and became a key instrument in mapping and sequencing genetic material.

1987: Applied Biosystems (USA) marketed the first automated sequencing machine, the model ABI 370. Constant improvements in the technology resulted in faster sequencing capacity, which was significant for advanced scientific research in projects such as mapping the human genome.

1990: The International Project on Human Genome was formally started and it was expected to take 15 years. It involved mainly the United States, the United Kingdom, France, Germany, Japan, China, and India.

1990: The US National Institutes of Health (NIH) began large-scale sequencing trials on *Mycoplasma* *capricolumn*, *Escherichia* *coli*, C*aenorhabditis* e*legans*, and *Saccharomyces* *cerevisiae*. 

1995: Craig Venter, Hamilton Smith and colleagues (USA) completed the first complete genome of a free-living organism, the bacterium *Haemophilus influenza*. The circular chromosome contains 1,830,137 bp [10]. This is the bacterium from which Smith had first isolated a restriction enzyme for which he shared the Nobel Prize in 1978.

1996: Applied Biosystems markets the capillary sequencer, ABI 310. It is an automatic single-capillary genetic analyzer designed for a wide range of sequencing and fragment analysis approaches.

1996: Pal Nyren and Mostafa Ronaghi (Sweden) developed the method of DNA pyrosequencing without the need for electrophoresis [11]. It relies on the detection of DNA polymerase activity by an enzymatic luminometric inorganic pyrophosphate detection assay developed by P. Nyren in 1987.

1998: Eric Kawashima, Laurent Farinelli and Pascal Mayer developed “Method of nucleic acid amplification” and obtained WO 98/44151 patent for its development [12] while working at the Glaxo Wellcome’s, Geneva Biomedical Research Institute. This method described DNA colony sequencing which is one of the milestones in developing the massive parallel sequencing technologies such as Illumina (San Diego, CA, USA) and Life Sciences 545 (a Roche Company, Branford, CT, USA).

1998: Phil Green and Brent Ewing (USA) developed the base calling program “Phred” for sequencer data analysis. It has the ability to estimate a probability of error for each base-call, as a function of certain parameters computed from the tracer data [13]. 

1998: Genome of the nematode C*aenorhabditis* e*legans* sequenced. The 97-megabase pair genomic sequence revealed over 19,000 genes. The complete genome sequence, the first from a multicellular organism and from an animal, was a joint sequencing project between the Welcome Trust Sanger Institute (Hinxton, UK) and the Genome Institute, Washington University, St. Louis, MO, USA.

2000: Human Genome Project: due to widespread international cooperation and advances in the field of genomics (especially in sequence analysis), as well as major advances in computing technology, a “rough draft” of the genome was finished in 2000 (announced jointly by U.S. President Bill Clinton and the British Prime Minister Tony Blair on June 26, 2000).

2001-2003: Human Genome Project: Key findings of the draft (2001) and essentially complete genome were announced in April 2003, 2 years earlier than planned (3.3 billion-base pairs; approximately 23,000 genes). 

## 4. The Need for Fast, Inexpensive and Accurate DNA Sequencing Technologies

The automated Sanger method had led to a number of major accomplishments, including the completion of the human genome and other selected animal and plant genomes. However, the method limitations showed a need for new and improved technologies for sequencing large numbers of human and other genomes. In the late 20th and early 21st century, efforts have been made towards the development of new methods to replace the automated Sanger method, which is considered as a “first-generation” technology. The newer methods are referred to as next-generation sequencing (NGS) and their use has changed the scientific approaches in both basic and applied research in many of scientific disciplines, especially in many branches of the biological field, including plant pathology and plant virology.

The major advance offered by NGS is the ability to produce an enormous volume of data, in several cases in excess of one billion short reads per instrument run, as well as its ability to deliver fast, inexpensive and accurate genome information. 

## 5. Development of NGS Platforms (2000–present)

In 2000, Massively Parallel Signature Sequencing (MPSS) Lynx Therapeutics (USA) Company launched the first of the NGS technologies. The company was later purchased by Illumina.

In 2004, 454 Life Sciences (Branford, CT, USA) marketed a paralleled version of pyrosequencing. The first version of their machine reduced sequencing costs 6-fold compared to automated Sanger sequencing and was the second of a new generation of sequencing technologies, after MPSS. Life Sciences acquired by Roche Company (Headquartered in Basel, Switzerland). Pyrosequencing provides intermediate read lengths and price per base compared to Sanger sequencing on the one hand and Illumina and SOLiD on the other. In 2005–2006, the 454 GS 20 Roche sequencing platform was introduced which revolutionized DNA sequencing as it could produce 20 million bases (20 Mbp). This was replaced by the GS FLX model in 2007 which is capable of producing over 100 Mbp of sequence in just four hours, which increased in 2008 to 400 Mbp. This model was then upgraded to the 454 GS−FLX+ Titanium sequencing platform which is capable of producing over 600 Mbp of sequence data in a single run with Sanger-like read lengths of up to 1,000 bp. Another sequencing platform system produced by Roche is the GS Junior which is small in size and is placed on a laboratory bench top. It provides long 400 bp sequencing reads with a fast sequencing run [14]. 

In 2005, Solexa released the Genome Analyzer (GA). Its sequencing technology is based on sequencing by synthesis (SBS) using reversible dye-terminators chemistry. Solexa was purchased by Illumina in 2007. The GAIIx platform generates up to 50 billion bases (50 Bbp) of usable data per run and the latest model can attain 85 Bbp per run. 

During the last 3–4 years, Illumina has developed the HiSeq platform series which include HiSeq® 2500, HiSeq 2000, HiSeq 1500 and HiSeq 1000 sequencing platforms. They vary in their outputs, run times, cluster generations, paired end reads, and maximum reads length with HiSeq 2500 the longest length of 200 bp and HiSeq 1000 the shortest one. HiSeq 2500 platform has the capacity to sequence a human genome in about 24 hours, “Genome in a day”. It may also sequence 20 exomes in a day, or 30 RNA sequencing samples in as little as five hours. It generates 120 billion b (120 Bbp) of data in 27 hours. Standard HiSeq 2000 generates 600 billion bases (600 Bbp) per run. The output of HiSeq 2500 can reach 600 Gbp. Illumina also released MiSeq in 2011, a bench top platform which shared most technologies with HiSeq series. It generates 1.5 Gbp per run in about 10 h [15]. 

SOLiD technology employs sequencing by oligo ligation detection. The result is sequences of quantities and length comparable to Illumina sequencing. SOLiD was purchased by Applied Biosystems (AB, founded in 2006 in Foster City, CA, USA and became a division of Life Technologies in 2008 Headquartered in Carlsbad, CA, USA). Owing to a two-base sequencing method, SOLiD accuracy may reach 99.99%. 

In 2007, the first SOLiD sequencing system was released by AB, followed by the SOLiD 5500 w and 5500 xlw sequencing systems in 2010. The SOLiD 5500 xlw has read lengths of 85 bp, with 99.99% accuracy and 30 Gbp per run. A complete run could be finished within a week. Applications of SOLiD include analysis of whole genome clusters. The SOLiD website, see [16], may be helpful. 

Other recently developed methods of NGS technologies include: Helicos sequencer released in 2009, Life Technologies Ion Torrent sequencer released in 2011 [17], Pacific Biosciences (Menlo Park, CA, USA) single molecule real-time (smrt) sequencer which also became available in 2011 [18], and Oxford Technologies Nanopore (Oxford, UK) single molecule sequencer with ultra long single molecule reads that became available in 2012–2013 [19]. In November 2012, Helicos Biosciences (Cambridge, MA, USA) filed for bankruptcy in Cambridge, MA, USA, and it is currently reorganizing its operation. For this reason its website is no longer available. 

Additional methods of NGS were also developed. These methods may include Polonator sequencing, Polony sequencing, DNA Nanoball sequencing, and VisiGen Biotechnologies sequencing.

Table 1 shows current major methods of NGS technologies and Table 2 shows current models of major sequencing platform systems and their applications in NGS technologies.

## 6. Platform Selection

Important factors in selecting a sequencing platform may include the size or expected size of the genome being studied, its complexity (including G+C content), and the depth of coverage and accuracy needed. Thus, it is important and advisable to contact the providers of the next-generation sequencing services for guidance. For *de novo* genome sequencing, longer read length may be appropriate. For fast turnover times and limited throughput, smaller laboratory bench top platforms may offer greater flexibility [20]. For amplicon sequencing, the Roche 454 platform is suitable because of its longer reads, however, it is currently expensive. Recently, the laboratory bench tops Roche 454 GS Junior, Illumina MiSeq, and Ion PGM have claimed that their sequencing platforms are suitable for sequencing amplicons. For RNA-seq and those projects that require high depths of coverage, Illumina and SOLiD platforms may offer the best all-round value for money, accuracy and throughput [21]. Roche 454 has the longest read length, Illumina HiSeq 2500 features the biggest output and lowest sequencing cost, and SOLiD 5500 xlw has the highest accuracy [22]. Table 3, Table 4, Table 5, Table 6, Table 7 and Table 8 show that during the last five years, Illumina sequencing platforms were used more frequently than those of Roche’s in different plant virology projects in several countries.

**Table 1:** Current major m1ethods of next-generation DNA sequencing technologies.

Sequencing platform	Amplification method	Sequencing chemistry	Read length (bp)	Sequencing Speed/h	Maximum Output Per run	Accuracy (%)	M^1^ I^2^ D^3^
454 (Roche)	Emulsion PCR	Pyrosequencing	400–700	13 Mbp	700 Mbp	99.9	0.10, 0.3, 0.02 [23]
Illumina (Illumina)	Bridge PCR	Reversible terminators	100–300	25 Mbp	600 Gbp	99.9	0.12, 0.004, 0.006 [23]
SOLiD (Life Technologies)	Emulsion PCR	Ligation	75–85	21–28 Mbp	80–360 Gbp	99.9	Error is higher than Illumina [24]
PacBio (Pacific Biosciences)	No amplification Single molecule real-time (or SMRT)	Fluorescently labeled nucleotides	4, 000–5,000	50–115 Mbp	200 Mb–1 Gbp	95	1, 2, 12 [25]
Helicos (Helicos Biosciences)	No amplification Single molecule	Reversible terminators	25–55	83 Mbp	35 Gbp	97	Error is in the range of few percent but higher than 454 and Illumina and biased toward InDels [24]
Ion Torrent (Life Technologies)	Emulsion PCR	Detection of released H	100–400	25 Mb–16 Gbp	100 Mb–64 Gbp	99	M, 0.06, I + D 1.38 [26]
Nanopore (Oxford Technologies)	No amplification Single molecule		Very long reads up to 50 kbp	150 Mbp	Tens of Gbp	96	

M ^1^ = Mismatch bases; I ^2^ =Insertion; D ^3^ = Deletion.

**Table 2:** Current models of major sequencing platform systems and their applications in next-generation sequencing technologies.

Platform Systems	Applications
454 GS FLX + Systems (GS FLX Titanium XL+/GS FLX Titanium XLR70) * GS Junior System (bench top)	DNA sequencing: whole genome sequencing, *de nov*o and resequencing of large genes in a single run with read length up to 1 kbp. Amplicon sequencing; RNA sequencing: transcriptome sequencing, sequencing capture metagenomics. Run time: 10–23 h; * Fast sequencing run with read length of 400 bp. Similar applications as the above system.
lllumina HiSeq Systems (2500/2000/1500/1000), Genome Analyzer IIx, HiScan SQ, * MiSeq (bench top)	DNA sequencing including candidate region targeted sequencing; Epigenetic sequencing: chromatin immunoprecipitation sequencing (ChIP-Seq), methylation analysis by sequencing; RNA sequencing: transcriptome analysis, small RNA and mRNA sequencing, gene expression profiling by sequencing; Run time: 8–14 days; * Up to 15 Gbp of output with 25 M sequencing reads and 2 × 300 bp read length; Access more sequencing applications such as exome, metagenomics, human leukocyte antigen (HLA) gene typing, mRNA sequencing, targeted gene expression (proteins and non-protein coding genes) such as rRNA, tRNA, or smRNA genes; Run time: 20–35 h.
SOLiD 5500 W Series Genetic Analysis Systems (5500 W, 5500 xlw)	DNA sequencing: whole genome and exome; Epigenetic Sequencing; RNA Sequencing. Run time: 7–12 days.
PacBio PACBIO RSII	DNA sequencing using single molecule real-time (SMRT) system with the longest read lengths of any sequencing technology. Characterization of genetic variation, methylation, targeted sequencing such as SNP detection and validation, indels, structural variants, haplotypes and phasing, base modification detection to understand gene expression, host-pathogen interactions, DNA damage and DNA repair. Run time: 30 min.
Helicos Genetic Analyzer System	DNA sequencing and RNA sequencing. Run time: 8 days.
Ion Torrent Ion PGM System (bench top) *Ion Proton System (bench top)	Semiconductor sequencing with 400-bp length- Ideal for sequencing small genes and genomes. DNA sequencing for microbial: genes and genomes, amplicons, exomes (unreveal disease-causing variants), targeted sequencing, viral typing and other microbial typing. RNA sequencing. Run time: 4.5 h * Semiconductor sequencing: Sequencing microbial genomes, exomes, transcriptomes. Run time: 4.5 h
Nanopore GridION System (bench top) *MinION System (a miniaturized disposable device for single use)	DNA sequencing; Epigenetic sequencing; Characterization of genetic variation. RNA sequencing: the system is designed to analyze the original sample RNA directly, without undergoing conversion to cDNA. Run time possibly under 60 min. * For DNA sequencing only, *i.e.*, blood DNA.

**Table 3:** Next-generation sequencing of plant viral siRNA, RNA or DNA from virus infected herbaceous, grass, fiber- producing, or vine hosts.

Host	Study finding/virome	Sample preparation/target	Sequencing platform	Ref.
Sweet potato	Detected*: Sweet potato feathery mottle virus*, *Sweet potato* chlorotic* stunt virus*; Discovered: two *Badnavirus* species (dsDNA), one *Mastrevirus* species (ssDNA)	siRNAs	Illumina	[27]
*Gomphrena globosa*	Plants were inoculated with unknown virus-a new *Cucumovirus* was identified. Proposed name: Gayfeather mild mottle virus	Total RNA + subtractive hybridization	Roche 454 GS-FLX	[28]
*Arabidopsis thaliana*	*Tobacco mosaic virus* siRNAs mediate virus-host interactions which may contribute to viral pathogenicity and host specificity	siRNAs	Illumina Genome Analyzer	[29]
*Nicotiana benthamiana*, *Arabidopsis thaliana*, *Cucumis milo* and tomato	Nine different viruses including *Cucumber mosaic virus*, *Tobacco rattle virus*, *Pepper mild mosaic virus*, *Potato virus X* were studied*.* The study extended the knowledge of distribution and composition of siRNAs in virus-infected plants and contributed to a better understanding of siRNAs biogenesis.	siRNAs of nine different viruses	Roche 454	[30]
Cassava	The complete sequence of the Tanzanian strain of *Cassava brown streak virus* was determined and compared with that of the Ugandan strain. The virus is highly heterogeneous at both the isolate and strain levels with nucleotide identity at the isolate level of 76%	Total RNA + subtractive hybridization	Roche 454 GS− FLX	[31]
*Nicotiana benthamiana*	Profiled *Cymbidium ringspot virus*-derived siRNAs. These RNAs were primarily produced from the positive strand of the virus, produced with different frequency, and had 5' monophosphate and were not perfect duplexes.	siRNAs	Roche 454 and Solexa (Illumina)	[32]
Wild plant species from 15 families naturally infected with viruses were utilized. The families are: *Acanthaceae*, *Bignoniaceae*, *Caesalpinaceae Commelinaceae*, *Cyperaceae*, *Cucurbitaceae*, *Euphorbiaceae*, *Gesneriaceae*, *Lamiaceae*, *Mimosaceae*, *Myrtaceae*, *Papilionaceae*, *Poaceae*, *Rubiaceae* and *Solanaceae*	Identification of 11 virus families in infected plants which include: *Bromoviridae*, *Caulimoviridae*, *Chrysoviridae*, *Closteroviridae*, *Endornaviridae*, *Luteoviridae*, *Narnaviridae*, *Partitiviridae*, *Potyviridae*, *Totiviridae*, and *Tymoviridae.* Unclassified virus families were also identified in some samples of infected plants. Discovered several thousand novel viruses, all linked to their specific plant hosts	dsRNAs	Roche 454-GS-FLX	[33]
Wild cocksfoot grass	*Cereal yellow dwarf virus* (*Luteovirus*) discovered infecting this grass (not previously reported infecting this host). *Cocksfoot streak virus* (*Potyvirus*) was detected	siRNAs	Roche 454	[34]
*Nicotiana benthamiana*, *Arabidopsis thaliana*	Profiling siRNAs of *Bamboo mosaic* *virus* and its interfering and non-interfering associated satellite RNAs. The overall composition of virus siRNAs and satellite RNAs in the infected plants reflect the combined action of virus, satellite RNA and different DCLs in host plants.	siRNAs (virus and satellites)	Solexa (Illumina)	[35]
Rice	Characterization of siRNAs of *Rice* *stripe virus* four genome RNAs in infected rice plants	siRNAs	Illumina Solexa	[36]
Cotton	Characterization of siRNAs of *Cotton* *leafroll dwarf virus* (genus *Polerovirus*, family *Luteoviridae*) in infected cotton plants	siRNAs	Illumina Genome Analyzer	[37]
Wild *Passiflora caerula* (Blue-crown passion flower) (a vine)	The complete nucleotide sequence of *the Passion fruit woodiness virus* (*Potyvirus*) was determined	Polyadenylated RNA	Illumina Solexa GAIIx	[38]
Pepper, eggplant	The complete nucleotide sequences of two new viruses *Pepper yellow leaf* *curl virus* (*Polerovirus*) and *Eggplant mild leaf mottle virus* (*Ipomovirus*) were determined	Purified virons viral RNA	SOLiD	[39]
Different hosts	A novel virus infecting watercress was identified (proposed name: Watercress white vein virus). Viruses such as *Piper yellow mottle virus*, *Arachacha* *virus B*, and *Potato black ring virus* were detected and sequenced	Partial virus and RNA purification then fragmented	Roche 454 GS−FLX	[40]
Tomato	siRNAs of *Tomato spotted wilt virus* were used to detect the virus in infected tomato before symptoms appeared at levels too low for conventional detection methods. Also, used for analysis of the virus quasi species and for identification of an unspecified *Tospoviru*s and a squash-infecting geminivirus	siRNAs	Illumina	[41]
*Arabidopsis thaliana*	Characterization of the siRNAs and transcriptome profiles of *Oilseed rape* *mosaic virus (Tobamovirus*)-infected *Arabidopsis* plants.	Total RNAs and siRNAs	Illumina Genome Analyzer	[42]
Tomato, *Nicotiana benthamiana*	Characterization of the siRNAs for the monopartite begomovirus *Tomato yellow leaf curl China virus* and its associated betasatellite in infected tomato and* Nicotiana benthamiana* plants . Also, it was found that the betasatellite affected the amount of virus siRNAs detected in both plant species.	siRNAs	Solexa-Illumina	[43]
Tomato	Identification of *Potato spindle tuber* *virod*, *Pepino mosaic virus*, differentiation of two strains of the virus, and a novel *Potyvirus* from infected tomato plants by obtaining the complete genome sequence of the mixed infected pathogens without prior knowledge of their existence. Based on the severity of symptoms on tomato, the novel virus is provisionally named “Tomato necrotic stunt virus”	siRNAs	Illumina Genome Analyzer IIx	[44]
Tobacco cv Xanthi nc	Identification of gene expression changes associated with disease development in tobacco plants induced by infection with the M strain of *Cucumber mosaic virus* Sequencing analysis of tobacco transcriptome identified 95,916 unigenes, 34,408 of which were new transcripts by database searches.	Total RNA then treated with DNase I	Illumina Hi Seq 2000	[45]
Corn (maize)	Detection and identification of *Maize chlorotic mottle virus* and *Sugarcane mosaic virus* by obtaining over 90% of both viral genomes sequencing which allowed also strain characterization. Next generation sequencing may be used for rapidly identifying potential disease causing agents, in this case viruses	Total RNA was purified from diseased tissue with virus symptoms	Roche 454 GS−FLX+	[46]
Seventeen plant species, among them 14 Australian indigenous species	Detection and identification of 12 viruses described previously members of the genera *Potyvirus*, *Nepovirus*, *Allexivirus and Carlavirus*. Four novel viruses were identified and proposed as members of the genera *Potyvirus*, *Sadwavirus and Trichovirus.* Moreover, in 3 cases, 2–3 distinct isolates of a virus species co-infected the same plant.	Polyadenylated RNA	Illumina Genome Analyzer IIx	[47]
*Nicotiana benthamiana*, *Laodelphgax striatellus (s*mall brown leafhopper), rice	The presence *Rice stripe virus* (RSV) siRNAs was demonstrated in infected rice as well as in infected *N. benthamiana* and viruliferous *L. striatellus.* Also, results indicate the potential existence of RNAi-mediated immunity against RSV infection in *L. striatellus*, a member of *Hemipteran* that transmits about 55% of the known plant viruses. Moreover, demonstrated that siRNA are generated differentially in different hosts.	siRNAs	Illumina	[48]
Sweet potato	Detected *Sweet potato feathery mottle virus* strain RC, *Sweet potato virus C* (*Potyvirus*), *Sweet potato chlorotic stunt virus* strain WA (*Crinivirus*), *Sweet potato leaf curl Georgia virus* (*Begomovirus*), and *Sweet potato* *pakakuy virus* strain B (synonym: *Sweet potato badnovirus* B) infecting sweet potato crops in Central America. Also, 4 viruses were detected in a sweet potato sample from the Galapagos Islands. Results suggest that siRNAs deep sequencing analysis is suitable for use as a reliable method for detection of plant viruses in infected crops.	si RNA	Illumina Genome Analyzer	[49]
Pepper (genotype Yolo Wonder)	Developing a mathematical model that estimates genetic drift and selection intensities using next generation sequencing data of 4 variants of *Potato virus Y* that differ by 1–2 substitutions involved in pathogenicity.	Total RNA	Roche 454	[50]
*Arabidopsis thaliana*	Analysis of viral siRNAs from DNA virus-infected cells showed that the entire circular genomes of *Cauliflower mosaic virus* (genus: *Caulimovirus*, family: *Caulimoviridae*) and *cabbage leaf curl virus* (genus: *Begomovirus*, family: *Geminiviridae*) are densely covered with siRNAs in both sense and antisense polarities without gaps. This would enable *de novo* reconstruction of the complete DNA virus genomes from siRNAs.	siRNAs	Illumina Genome Analyzer	[51,52,53]
Black pepper	The complete genome sequence of *Piper yellow mosaic virus* was determined. It was also established that the virus is a member of the genus *Badnovirus* and the family *Caulimoviridae.* Fragments of two additional novel viruses belong to *Caulimoviridae* were sequenced and the viruses were tentatively named Piper DNA virus 1 and 2.	Viral and plant DNA were isolated from virus-enriched fraction	Roche 454 GS−FLX Titanium	[54]

**Table 4:** Next-generation sequencing of plant viral siRNA, total RNA or dsRNA from virus-infected temperate fruit crop, citrus or fig hosts.

Host	Study finding/virome	Sample preparation/target	Sequencing platform	Ref.
Raspberry	A novel virus isolated from infected raspberry plants was completely sequenced and characterized. It was designated as *Raspberry latent virus*. The virus is a novel dicot-infecting reovirus in the family *Reoviridae*, subfamily *Spinareovirinae.*	dsRNA	Illumina	[55]
Citrus	In *Citrus tristeza virus* (CTV) infected citrus plants It was shown that the citrus homologues of Dicer-like ribonucleases mediate the genesis of the 21 and 22 nt CTV siRNAs and that the ribonucleases act not only on the genomic RNA but also on the 30 co-terminal subgenomic RNAs and, particularly, on their dsRNA forms. A novel citrus miRNAs was also indentified and how CTV influences their accumulation was determined.	CTV sRNAs, gRNA sgRNAs	Illumina	[56]
Citrus	Genomic organization and other molecular characterizations were determined for *Citrus yellow vein clearing virus.* Analyses suggested that the virus is the causal agent of yellow vein clearing disease of lemon trees and represent a new species in the genus *Mandarivirus.*	siRNAs	Illumina	[57]
Citrus	A novel DNA virus species, member of the family *Geminiviridae*, was identified and associated with citrus chlorotic dwarf disease. A provisional name of Citrus chlorotic dwarf-associated virus was proposed.	siRNAs and total DNA	Illumina HiSeq2000	[58]
Apple, Citrus, Grapevine	Detected ASPV, ACLSV and an unknown mycovirus. Detected two variants of CTV and ASGV. Detected variants of GLRaV-3, GVA and an unknown mycovirus.	siRNAs	Illumina	[59]
Apple	Identified agents associated with green crinkle disease of apple trees. The disease is a complex one as the following viruses were identified associated with it: ASGV, ASPV, ACLSV, ApLV, ApPCLSV and PCMV.	siRNAs	Illumina HiSeq2000	[60]
*Prunus*	Detected and identified known *Prunus* viruses such as PPV, PNRS, etc. and novel virus agents.	dsRNA	Roche 454	[61]
Fig	Detected *Fig mosaic virus* and *Fig* *latent virus*-1 for their elimination from infected clones. It is the first application of next-generation sequencing technology to detect and identify known and new species of viruses infecting fig trees.	dsRNAs	Illumina	[62]
Blackberry	A novel *Ampelovirus* in the family *Closteroviridae* was identified as one of the viruses associated with blackberry yellow vein disease complex.	dsRNAs	Illumina	[63]
Cherry	Characterization of the genome of the divergent *Little cherry virus* 1 (LChV1) isolate and establishing that LChV1 isolates could be responsible for Shirofugen stunt disease syndrome.	dsRNAs	Roche 454 Pyrosequencing multiplex approach	[64]
Citrus	The complete nucleotide sequence and structure of a novel virus of the genus *Cilevirus* was determined. The novel virus causes symptoms similar to citrus leprosies and it is suggested to be called Citrus leprosis virus cytoplasmic type 2.	siRNAs	Illumina	[65]
Citrus	A novel virus was discovered by analysis of the contigs assembled from the virus siRNAs sequences which showed similarity with luteovirus sequence, particularly with *Pea* *enation mosaic virus*, the type member of the genus *Enarnovirus*. The complete genome of the virus was determined and the new virus was provisionally named Citrus vein enation virus.	siRNAs	Solexa-Illumina	[66]

Virus abbreviations: ACLSV, *Apple chlorotic leaf spot virus*; ApLV, *Apricot latent virus*; ApPCLSV, *Apricot pseudo-chlorotic leaf spot virus*; ASGV, *Apple stem* *grooving virus*; ASPV, *Apple stem pitting virus*; CTV, *Citrus tristeza virus*; GLRaV-3, *Grapevine leaf roll associated virus* 3; GVA, *Grapevine virus A*; PCMV, *Peach chlorotic mottle virus*; PNRS, *Prunus necrotic ring spot virus*; PPV, *Plum pox virus.*

**Table 5:** Next-generation sequencing of plant viral siRNA, total RNA or dsRNA from virus-infected grapevine.

Host	Study finding/virome	Sample preparation/target	Sequencing platform	Ref.
Grapevine	A novel *Marafivirus* (*Grapevine Syrah* 1*virus*) was identified associated with grapevine syrah decline. The virus was also identified in leafhopper vector. Also detected in plant tissue GRSPaV, GRVFV, GLRaV-9, and viroids.	Total RNA or dsRNA	Roche 454	[67]
Grapevine	*Grapevine virus* E, not previously in South Africa. A mycovirus similar to *Penicillium chrysogenum virus*, two other mycoviruses, GLRaV-3, GRSPaV, GVA.	dsRNA	Illumina	[68]
Grapevine	Viruses of the genera *Foveavirus*, *Maculavirus*, *Marafivirus*, and *Nepovirus* were detected. siRNAs originate from both genomic and antigenomic strands with the exception of tymoviruses, the majority are derived from antigenic virus strand.	siRNAs	Illumina	[69]
Grapevine	A novel DNA virus was discovered associated with the grapevine vein-clearing and vine decline syndrome. The virus belongs to genus *Badnavirus* in the family *Caulimoviridae.* It is the first DNA virus discovered in grapevine. It has been provisionally named Grapevine vein clearing virus.	siRNAs	Illumina Genome Analyzer	[70]
Grapevine	Twenty six fungal groups were identified in a single plant source. Three of the mycoviruses were associated with *Botrytis cinerea*. Most of the rest were undescribed.	dsRNA	Roche 454	[71]
Grapevine	A novel species of virus was discovered for which the provisional name Grapevine Pinot gris virus is proposed. Also, detected and identified GRSPaV, GRVFV, GSy 1V, and viroids.	siRNAs	Illumina	[72]
Grapevine	A novel circular DNA virus was identified associated with red blotch disease in grapevine in California. A provisional name of Grapevine red blotch-associated virus is proposed for the novel virus.	dsRNA extracted without DNase treatment	Illumina Genome Analyzer IIx	[73]
Grapevine	A novel *Vitivirus* was identified. The virus is provisionally named Grapevine virus F.	dsRNA	Illumina Genome Analyzer IIx	[74]
Grapevine	Characterization of siRNAs associated with grapevine leafroll disease.	siRNAs	Illumina	[75]
Grapevine	Complete sequence of a novel single-stranded DNA virus associated with grapevine red leaf disease (GRD). The virus is tentatively named Grapevine red leaf-associated virus (GRLaV). The virus represents an evolutionary distinct lineage in the family *Geminiviridae*. Also detected in plant tissue GRSPaV, GFV, and viroids.	Total RNA treated with DNase	Illumina Genome Analyzer IIx	[76]

Virus abbreviations: GFV, *Grapevine fanleaf virus*; GLRaV-3, *Grapevine leaf roll-associated virus* 3; GLRaV-9, *Grapevine leaf roll-associated virus* 9; GRSPaV, *Grapevine rupestris stem pitting-associated virus*; GRVFV, *Grapevine rupestris vein feathering virus*; GSy 1V, *Grapevine Syrah* 1*virus*; GVA, *Grapevine virus* A.

**Table 6:** Next-generation sequencing of isolated siRNA or dsRNA from viroid-infected grapevine, peach, cucumber, *Nicotiana benthamiana* or tomato hosts.

Host	Study finding/virome	Sample preparation/target	Sequencing platform	Ref.
Grapevine	Different Dicer-like enzymes target RNAs of *Hop stunt viroid*, *Grapevine* *yellow speckle viroid* 1. Also, study suggested that the viroid RNAs may interact with host enzymes involved in the RNA-directed DNA methylation pathway	siRNAs	Solexa, Illumina	[77]
Grapevine	Detection and identification of *Australian grapevine viroid*, *Hop stunt* *viroid* and *Grapevine yellow speckle viroid*	Total RNA or dsRNA	Roche 454	[67]
Peach	To study the genesis of *Peach latent mosaic viroid* siRNA and viroid pathogenesis	siRNAs	Illumina	[78]
*Nicotiana* *benthamiana*	RNA-dependent RNA polymerase 6 restricts accumulation and precludes meristem invasion of *Potato spindle tuber viroid* which replicates in nuclei	Plant and viroid siRNAs	Illumina EAS269 GAII	[79]
*Cucumber*	To study *Hop stunt viroid* pathway involved in the biogenesis of the viroid siRNAs	siRNAs	Illumina	[80]
Tomato	Detection and identification of *Potato spindle tuber viroid*	siRNAs	Illumina Genome Analyzer IIx	[44]
Grapevine	Detection and identification of *Grapevine yellow speckle viroid* 1 and *Hop stunt viroid*	siRNAs	Illumina	[72]
Grapevine	Detection and identification of *Grapevine yellow speckle viroid* 1 and *Hop stunt viroid*	siRNAs and dsRNAs	Illumina	[81]
Grapevine	Discovery of viroid-like circular RNA 375 nt long with hammerhead ribozymes. Currently, infectivity studies showed that the RNA is not infectious which may suggest that it is viral satellite	siRNAs	Illumina	[82]
Grapevine	Characterization of siRNAs of *Hop stunt viroid*, *Grapevine yellow speckle* *viroid* 1, and *Grapevine yellow speckle viroid* 2	siRNAs	Illumina	[75]
Grapevine	Detection and identification of *Grapevine yellow speckle viroid 1*, *Hop stunt viroid*, *Citrus exocortis* *Yucatan viroid* and *Citrus exocortis viroid* from both symptomatic and non-symptomatic samples of grapevine read leaf disease	Total RNA treated with Dnase	Illumina Genome Analyzer IIx	[76]

**Table 7:** Next-generation sequencing of RNA viruses, DNA viruses or bacteria DNA from insect vectors.

Vector	Study finding/virome	Sample preparation/target	Sequencing platform	Ref.
Grapevine leafhopper	A novel *Merafivirus* associated with grapevine syrah decline was detected in the vector	Total nucleic acids of the viruliferous vector	Roche 454	[67]
Citrus psyllid	A complete genomic sequence of the bacterium, “Candidatus Liberibacter asiaticus” was obtained. The genome is circular and its size is about 1.23 Mb, The bacterium is the causal agent of citrus Huanglongbing (greening) disease	DNA extracted from a single “Ca. L. asiaticus”-infected Asian citrus psyllid (*Diaphorina citri*)	Roche 454 GS−FLX	[83]
*Bemesia tabaci*	Four novel *Begomovirus* species were discovered in their viruliferous vectors	Purified viral DNA	Metagenomic reads 100–700 nt	[84]
*Aodelphgax striatellus* (small brown leafhopper)	The presence of *Rice stripe virus* siRNAs was demonstrated in the viruliferous vector *L. striatellus*	siRNAs	Illumina	[48]

**Table 8:** Next-generation sequencing of isolated DNA, RNA or miRNA from grapevine, periwinkle or citrus infected with phytoplasmas or difficult to culture bacteria.

Host	Study finding/pathogen	Sample preparation/target	Sequencing platform	Ref.
Grapevine	It was demonstrated that sequences of infected phytoplasmas belonged to 16 SrV and 16 SrXII groups, as well as to *Candidatus* Phytoplasma prunorum (16SrX-B) whereas some sequences could not be assigned to a single phytoplasma group. Also a high number of single nucleotide polymorphisms (SNPs) were found. Suggested NGS may be used for future phytoplasma detection in quarantine.	Total DNA from mid-vein leaf tissue	Amplicon sequencing by Roche 454 GS FLX	[85,86]
Grapevine	Demonstrated significant changes in the transcriptome of Aster yellows phytoplama-infected Grapevine cv. Chardonnay. The study could contribute to understanding the unknown mechanisms of phytoplasma pathogenicity.	Total RNA and DNA	Illumina HiSeq 2000	[87]
Periwinkle	Genomic analysis of four phytoplasma strains of 16SrIII group and two strains of the 16SrI-B subgroup revealed the significant role of horizontal gene transfer among different “Ca. Phytoplasma” species in shaping phytoplasma genomes and promoting their diversity.	Standard DNA preparation from infected periwinkle	Illumina	[88]
Citrus (Mexican lime)	Identified miRNA families that are expressed differentially upon infection of Mexican lime trees with Candidatus Phytoplasma aurantifolia. The study increases our understanding of the molecular basis of witches’ broom disease which may lead to development of new strategies for its control.	miRNAs were isolated from infected and from healthy tissues	Illumina HiSeq 2000	[89]
Citrus	Demonstrated that several miRNAs and siRNAs were highly induced by Ca. L. Asiaticus (Las) infection, which can be potentially developed into early diagnosis markers of huanglongbing (HLB) disease (citrus greening disease). MiR399 was induced specifically by infection of Las. MiR399 is induced by phosphorous starvation in other plant species. Applying phosphorous significantly reduced HLB symptoms in citrus.	RNA fragments 18–28 nt in length obtained after fractionation of total RNA on denaturing polyacrylamide gel electrophoresis. The total RNA was extracted from Las-infected tissue with HLB symptoms	Illumina	[90]

## 7. Bioinformatics’ Software Tools for Data Analysis

The software functions for next-generation sequencing data analysis may be classified into four general categories which include alignment of sequence reads, base-calling and/or polymorphism detection, *de* *novo*, and genome browsing and annotation. Several software packages have been developed for each category. For example, a variety of bioinformatics’ software tools for short-read sequencing used for *de novo* assembly of genomes and transcriptomes are available [91,92]. A practical application of *de* *novo* genome assembly software tools has been also recently reported [93]. New categories and new software tools for short-read sequencing analysis are continuously being developed commercially world-wide, especially in the U.S., Europe and Australia. Many commercial sequencing companies, *i.e.*, Roche, Illumina, SOLiD, *etc*., offer their software services to customers for different types of DNA or RNA sequencing analysis. It is worth mentioning that alignment software such as BLAST or BLAT are not suitable for short-read sequencing analysis of next generation sequencing as they were developed specifically for long reads generated by conventional first generation sequencing.

A full review of software tools available for quality control assembly and quantitative analysis of next-generation sequencing is beyond the scope of this article, but have been the subject of several review articles, *i.e.*, [94,95,96,97] and a book [98]. Moreover, the journal *Bioinformatics* devoted one of its volumes published in 2009 (volume 25, No.4) to bioinformatics tools and algorithms that have been developed for next-generation sequencing data analysis. These bioinformatics software tools and algorithms are continuously being developed and improved to keep abreast and pace with the latest advances of next-generation sequencing technologies.

## 8. Cost of DNA Sequencing

**Figure 1:**
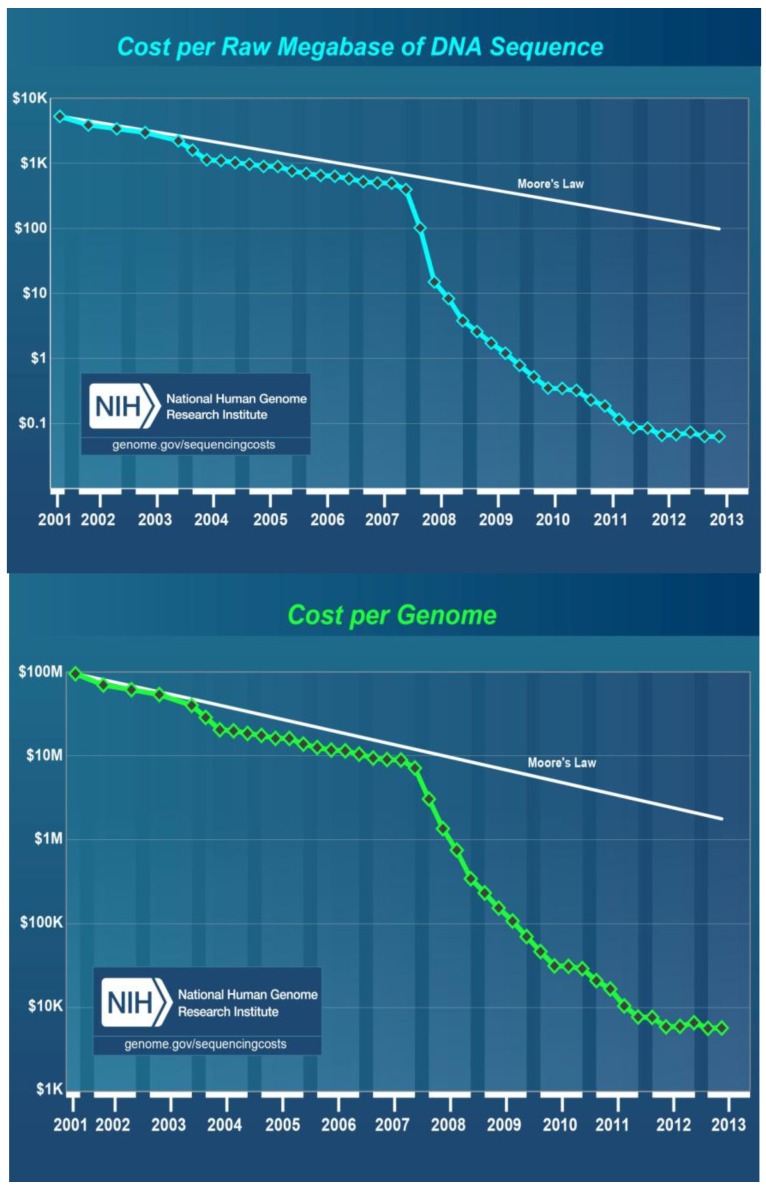
Cost in US Dollars per raw megabase of DNA sequence (**top**) and cost per genome (**bottom**) from July 2001 to July 2013 as estimated by the National Human Genome Research Institute, U.S. National Institues of Health, Bethesda, MD, USA. During this time period, first generation sequencing methods were used from 2001 through 2007, then NGS platforms were used from 2008 to present.

According to the latest information released by the National Human Genome Research Institute, U.S. National Institues of Health, the cost per raw megabase of DNA sequence and the cost per genome were reduced dramatically from July 2001 to July 2013 [99]. During this time period, first generation sequencing methods were used from 2001 through 2007, then, second generation NGS platforms were used from 2008 to present, 2013. The cost per raw megabase of DNA sequence was reduced from about $8,000 in 2001 to $700 in 2007 and then to less than $0.1 in 2013 (Figure 1, top). Similarly, the cost per genome DNA sequence went from $100, 000,000 in 2001 to $10,000,000 in 2007 then to about $8,000 in 2013 (Figure 1, bottom).

In the next few years, it is expected that the third generation of DNA sequencing platforms will increase sequencing capacity and speed while reducing cost. A genome may be sequenced for about $1,000 or less in about 15 minutes. That is 85% less than the current cost. 

## 9. Biological Applications of Next-Generation Sequencing

To date the biological applications of next-generation sequencing focused primarily on:

Full or complete genome sequencing (*de novo* sequencing and/or resequencing genomics): Its aim is to sequence the entire genome of an organism such as humans, primates, dogs, cats, mice, nematodes, fungi, bacteria, viruses, *etc*. It is worth noting that unlike full genome sequencing, DNA profiling only determines the likelihood that genetic material came from a particular individual or group; single nucleotide polymorphism (SNP) genotyping covers less than 0.1% of the genome.

Metagenomics: The term “metagenomics” was first used in publications in 1998. Consequently in 2005 Kevin Chen and Lior Pachter at University of California (Berkeley, CA, USA), defined metagenomics as “the application of modern genomics techniques to the study of communities of microbial organisms directly in their natural environments, by passing the need for isolation and lab cultivation of individual species.” The goals of this approach are to characterize organisms present in a sample and identify what roles each organism has within a specific natural environment. Metagenomics microbial samples are found almost everywhere such as the human body, soil samples, plant samples, water samples, *etc*.

Transcriptome sequencing: It includes sequencing and analysis of full-length mRNA and small RNA (microRNA profiling and discovery); mRNA transcript expression analysis (full-length mRNA, expressed sequence tags [ESTs] and ditags, and allele-specific expression); quantification of gene expression and alternative splicing, transcript annotation, discovery of transcribed SNPs or somatic mutation.

Sequencing of the cDNAs of diverse RNAs (genomic, mRNA, micro RNA, ribosomal RNA, *etc*.) has been coined RNA-seq. [100].

Amplicon sequencing: This method allows the detection of mutations at extremely low frequency levels in PCR amplified specific targeted regions of DNA. It has been used to identify low frequency somatic mutations in cancer samples, discovery of rare variants in HIV-infected patients, *etc*. It also has been used recently for sequencing phytoplasmas from grapevines.

Other applications of NGS in specific categories may include reduced representation sequencing, (*i.e*., large-scale polymorphism discovery), targeted genomic resequencing, (*i.e.*, targeted polymorphism and mutation discovery), paired end sequencing, (*i.e.*, discovery of inherited and acquired structural variation), sequencing of bisulfite-treated DNA, (*i.e*., for determining patterns of cytosine methylation in genomic DNA), chromatin immunoprecipitation sequencing (ChIP-Seq), (*i.e.*, genome-wide mapping of protein-DNA interactions) , nuclease fragmentation and sequencing, (*i.e.*, nucleosome positioning) [92]. In addition, other applications include: pharmogenomics sequenceing to determine suitable drugs for individuals; diagnosis and discovery of new human diseases; cancer therapy; humans origin, migration of humans, evolution, and others. 

## 10. Applications of Next-Generation Sequencing in Medical Virology

The first applications of next-generation sequencing in the field of medical virology were to monitor population diversity in HIV [101,102]. In September 2008, an outbreak of unexplained hemorrhagic fever in people was reported in South Africa which resulted in death of four out of five patients. Such outbreaks required a rapid response to control both infection and public anxiety. NGS of infected blood serum samples revealed the discovery of a novel *arenavirus* [103]. Rapid pathogen discovery using NGS is now available to monitor all outbreaks, regardless of the disease.

Currently, next-generation sequence technologies applications in medical virology may include, but are not limited to, determination of: full-length viral genome sequence; study of viral genome variability; characterization of viral quasi-species; viral evolution. Metagenomics-based strategies have been used for detection of unknown viral pathogens, discovery of novel viruses, detection and identification of tumor viruses; characterization of the human “viral metagenome” or “virome” in healthy and disease conditions and epidemiology of viral infection. These strategies have also been used for analysis of resistance profiles to drugs and host immunity; quality control of viral vaccines; transcriptomics studies for measurements of mRNA to achieve new insights into genome expression and how this may be modified in health and disease as well as for exploring the role of microRNAs in virus replication and pathogenesis (for review, see [21,104]). 

## 11. Current Applications of Next-Generation Sequencing in Plant Virology

NGS technologies combined with sophisticated bioinformatics have been recently changing the field of plant virology, particularly in the areas of genome sequencing, ecology, discovery, epidemiology, transcriptomics, replication, detection and identification. Currently a small number of plant RNA viruses and viroids have been identified from infected tissues and sequenced by RNA-seq. Either total nucleic acid or total double-stranded RNA (dsRNA) from pathogen infected tissue was isolated and the virus or viroid was identified by NGS; alternatively, in some cases, host nucleic acid was partially eliminated by hybridization to nucleic acid isolated from healthy plants to enrich for virus sequences in the infected plant material prior to sequencing. All the viruses and/or viroids detected and identified in infected plants or viruses in viruliferous vectors using NGS are termed “virome”.

Plant viruses or viroids can also be detected indirectly. In response to infection by RNA/DNA viruses or viroids, the host plant generates specific RNA molecules, 21 to 24 nt in length, called short interfering RNAs (siRNA). RNA silencing (RNAi) is a cytoplasmic cell surveillance system to recognize dsRNA and specifically destroy single and double stranded RNA molecules homologous to the inducer, using small interfering RNAs as a guide (for review see [105,106]). NGS of siRNAs offers good opportunities to identify viruses or viroids infecting plants, even at extremely low titers, in symptomless infections, and including previously unknown viruses or viroids. Next-generation sequencing can provide thousands to millions of siRNA sequences from virus or viroid infected plant materials. When virus or viroid-derived siRNAs are abundant enough, virus or viroid genome fragments can be assembled. Since the virus or viroid siRNAs are 21–24 nt in length, their sequences can be employed directly as primer sequences to amplify viral or viroid fragments by PCR or RT-PCR. 

Table 3, Table 4, Table 5 and Table 6 summarize the use of next generation technologies in analyzing virus or viroid virome in infected plant species. NGS technologies have also been used for detection and identification of virus virome in viruliferous vectors (Table 7) and insect viruses [107], and the bacterial “virome” of the bacterium ‘Candidatus Liberibacter asiaticus’ in its insect vector (Table 7) and infected host plant (Table 8). In addition, these technologies have been used for analyzing phytoplasma “virome” in infected plants (Table 8). 

## 12. Next-Generation Sequencing and Revealing the Etiology of Unknown Diseases and Latent Infections

The recent use of NGS technologies in plant virology revealed that some diseases of unknown etiology that affect herbaceous and grass hosts or latent agent infections in wild hosts of different species are caused by new or known viruses (Table 3). This table shows that the following novel viruses were discovered: (a) in sweet potato, two dsDNA viruses (*Badnavirus*) and one ssDNA virus (*Mastrevirus*); (b) in *Gomphrena globosa* mechanically inoculated with sap from *Liatris spicata*, a *Cucumovirus* provisionally named Gayfeather mild mottle virus; (c) in wild plant species from 15 families, unclassified virus families and numerous novel virus species; (d) in wild cocksfoot grass, *Cereal dwarf virus* (*Luteovirus*) discovered infecting this plant species; (e) in pepper and eggplant, *Pepper yellow leaf curl virus* (*Polerovirus*) and *Eggplant mild leaf mottle* (*Ipomovirus*), respectively; (f) watercress, a virus provisionally named Watercress white vein virus; (g) in tomato, a *Potyvirus* species with the proposed name Tomato necrotic stunt virus, also in tomato in a different study, an unspecified *Tospovirus* and a squash-infecting geminivirus; (h) in 17 wild plant species among them 14 Australian indigineous species, four novel viruses (*Potyvirus*, *Sadwavirus*, *Trichovirus*); (i) in black pepper, two DNA viruses (*Caulimoviridae*) with proposed names DNA virus 1 and DNA virus 2.

Most, if not all, diseases of unknown etiology that affect stone, pome and small fruit crops, as well as citrus, and grapevine are graft transmissible. That may imply that systemic pathogens are most likely involved in the etiology of these diseases. Currently there are over 35 diseases of unknown etiology that affect stone fruits and a similar number that affect pome fruits [108]. As revealed recently by next generation sequencing, the disease apple green crinkle is a disease complex and at least 6 known viruses—the 3 apple latent viruses and 3 stone fruit viruses—are associated with it (Table 4). Also, it was established that Shirofugen stunt disease syndrome could be caused by *Little cherry virus* 1 (Table 4). Among major diseases of unknown etiology that affect small fruits are blueberry fruit drop, blueberry bronze leaf curl, blackberry yellow vein, raspberry leaf curl, raspberry yellow spot and strawberry latent C [109]. Quito-Avila *et al*. [55] discovered a novel reovirus in the family *Reoviridae*, subfamily *Spinareovirinae* infecting raspberry; Thekke-Veetil *et al*. [63] identified a novel *Ampelovirus* as one of the viruses associated with blackberry yellow vein disease (Table 4).

In citrus, a novel DNA geminivirus was discovered and provisionally named Citrus chlorotic dwarf-associated virus; two novel RNA viruses were also discovered, one was provisionally named Citrus leprosies virus cytoplasmic type 2 (*Cilevirus*) and the other was named Citrus vein enation virus (*Enamovirus*) (Table 4).

NGS has also been used for detection and identification of virus virome in viruliferous vectors as well as of the bacterium, “Candidatus Liberibacter asiaticus” in its vector (Table 7). It has also been utilized for the detection and discovery of insect viruses [107].

In addition to viruses and viroids, NGS technologies have been used for analyzing phytoplama “virome” in infected grapevine, periwinkle or citrus (Table 8). Total DNA and/or RNA or siRNAs from infected host were used in these studies. Moreover, it was also demonstrated that, upon citrus infection by the hard to grow bacterium, “Candidatus Liberibacter asiaticus”, the causal agent of citrus Huanglongbing (greening) disease, several miRNAs and siRNAs were highly induced and can be potentially developed into early diagnosis markers of the disease (Table 8).

Next generation sequencing has been shown to be a good option for investigating diseases of unknown etiology in grapevine [81]. A novel *Marafivirus* (*Grapevine Syrah* 1 *virus*) was identified associated with grapevine syrah decline (Table 5). In addition, the following novel RNA viruses were discovered infecting grapevine and given the provisional names: Grapevine Pinto gris virus, Grapevine virus F. (*Vitivirus*). Two DNA viruses were also discovered, the first belongs to the genus *Badnovirus*, family *Caulimoviridae,* provisionally named Grapevine vein clearing virus; the second is a DNA geminivirus and given the provisional name Grapevine red-blotch-associated virus or Grapevine red-leaf-associated virus (Table 5). A viroid-like circular RNA with a hammerhead structure was discovered in infected grapevine (Table 6). Currently, infectivity studies showed that the RNA is not infectious which may suggest it is a viral satellite.

## 13. Prospective

NGS technologies have become available during the last several years and have been widely used in whole genome sequencing, metagenomics, RNA sequencing, *etc*. The technologies are in continuous development and improvement. Sequencing chemistries are becoming established, maturing and evolving, read lengths and fidelity are increasing, allowing us to investigate thoroughly viral genome diversity, virus-host interaction, virus epidemiology, virus diagnosis and elimination, *etc*. The major sequencing platforms are being developed to be easier to use and more cost effective. Moreover, very recently Roche, Illumina, Life Technologies Ion Torrent and Oxford Technologies Nanopore sequencing companies have developed and released in the market reasonably priced bench top platforms with the aim of making next-generation sequencing technologies more readily available to more research laboratories as well as to diagnostic laboratories for viral, bacterial and fungal human, animal, and plant diseases. In 2012–2013 single-molecule DNA sequencing in a miniaturized disposable device for single use has been developed by Oxford Technologies Nanopore that allows simple and cheap high throughput sequencing. It is worth mentioning that Nanopore sequencing platform models have the potential to rapidly generate ultra long single molecule reads.

The most recent report of the International Committee for the Taxonomy of Viruses lists about 900 plant viruses [110]. The utilization of NGS in plant virology will definitely increase this number very significantly as new viruses are being discovered and characterized in different plant host species, including wild ones, as well as in different insect vectors. Conventional serological or molecular detection and identification methods of plant viruses or viroids depend on prior knowledge of antibody or sequence of the virus or viroid of interest. The NGS technologies have provided a very powerful alternative for detection and identification of these pathogens without a *priori* knowledge. Metagenomics developed by NGS technologies has been proven to be sensitive, accurate and fast in detection and identification of known and unknown viral and viroid viromes without bias in infected plant species, including woody perennial crops which have low titers of these pathogens. Thus, NGS technologies have the potential to be used in quarantine and certification programs of grapevine, pome and stone fruits, small fruits as well as citrus. The potential use of these technologies in diagnostics has been recently suggested in temperate fruit crops [111], citrus [112], grapevine and other crops [113]. Data generated by these technologies can be used effectively to improve efficiency and reliability of these programs as well as in programs aimed at virus and viroid elimination from vegetatively propagative material. Accordingly, NGS technologies will be a significant and powerful tool in controlling virus and viroid diseases. 

## 14. Conclusions

The next-generation high throughput sequencing technologies have been available for several years. These technologies and bioinformatics provide rapid and low cost DNA sequencing for biological material, including plant viruses and viroids. NGS platforms, which have different underlying biochemistries and differ in sequencing protocol, produce large amounts (typically millions to billions) of generally short DNA sequence reads, length typically between 25 bp and 400 bp. These reads are shorter than the 750 bp first-generation Sanger sequences. The beginning of the era of NGS technologies was in 2000 and the first marketed sequencing platform was in 2004. Since then the NGS industry has expanded with several companies marketing different models of sequencing platforms, most recently the bench top platforms. The sequencing platforms are continually improved to become faster, more efficient and cheaper in order to bring next-sequencing technologies into many more laboratories to expand their use in biological research and diagnostics. 

NGS technologies combined with sophisticated bioinformatics were successfully utilized in plant virology since 2009, particularly in the areas of genome sequencing, detection and identification, discovery, transcriptomics, replication, ecology, and epidemiology. Known and novel plant RNA and DNA viruses and their satellites as well as viroids and phytoplasmas from different infected plant species were successfully studied using these technologies. The number of published studies, however, are still small but significant (Table 3, Table 4, Table 5, Table 6, Table 7 and Table 8). The utilization of NGS in plant virology in the near future will definitely increase whether in research or diagnostics. The potential use of these technologies in plant certification and quarantine programs can effectively improve the efficiency and reliability of these programs and in controlling virus and viroid diseases at both the national and international levels. 
